# The structure and Hirshfeld surface analysis of the salt 3-methacryl­amido-*N*,*N*,*N*-tri­methyl­propan-1-aminium 2-acryl­amido-2-methyl­propane-1-sulfonate

**DOI:** 10.1107/S2056989019012003

**Published:** 2019-09-10

**Authors:** Ravindra N. Wickramasinhage, C. John McAdam, Lyall R. Hanton, Stephen C. Moratti, Jim Simpson

**Affiliations:** a Department of Chemistry, University of Otago, PO Box 56, Dunedin, New Zealand

**Keywords:** crystal structure, 3-methacryl­amido-*N*,*N*,*N*-tri­methyl­propan-1-aminium, 2-acryl­amido-2-methyl­propane-1-sulfonate, hydrogen bonding, Hirshfeld surface analysis

## Abstract

The title salt, C_10_H_21_N_2_O^+^·C_7_H_12_NO_4_S^−^, comprises a 3-methacryl­amido-*N*,*N*,*N*-tri­methyl­propan-1-aminium cation and a 2-acryl­amido-2-methyl­propane-1-sulfonate anion. The salt crystallizes with two unique cation–anion pairs in the asymmetric unit of the ortho­rhom­bic unit cell. The crystal studied was an inversion twin with a 0.52 (4):0.48 (4) domain ratio. In the crystal, the cations and anions stack along the *b*-axis direction and are linked by an extensive series of N—H⋯O and C—H⋯O hydrogen bonds, forming a three-dimensional network. Hirshfeld surface analysis was carried out on both the asymmetric unit and the two individual salts. The contribution of inter­atomic contacts to the surfaces of the individual cations and anions are also compared.

## Abstract 2

The mol­ecular and crystal structure of the salt 3-methacryl­amido-*N*,*N*,*N*-tri­methyl­propan-1-aminium 2-acryl­amido-2-methyl­propane-1-sulfonate, that crystallizes with two unique pairs of cations and anions in the asymmetric unit, is reported. Hirshfeld surface analysis of the asymmetric unit and of the two individual salts is also carried out.

## Chemical context   

We are currently inter­ested in tough hydro­gels with a built-in capacity for self-healing, as a means of improving their performance in practical applications (Goswami *et al.*, 2017 ▸; Pushparajan *et al.*, 2018 ▸). One approach involves the polymerization of ion-pair comonomers (IPC) typically based on sulfonate anions and quaternary ammonium cations (McAdam *et al.*, 2019 ▸). The covalent cross-linking of mixed cationic and anionic monomers generates polyampholytes (Zurick & Bernards, 2014 ▸) with additional toughness and self-healing ability due to electrostatic inter­actions between the oppositely charged functional groups present (Ihsan *et al.*, 2016 ▸; Haag & Bernards, 2017 ▸). The title IPC salt was first reported in 1978 at the emergence of this field (Salamone *et al.*, 1978 ▸). The original synthesis utilized ion-exchange chromatography (Salamone *et al.*, 1980 ▸) but this preparative methodology has been superseded by the argentometric mixing approach (Li *et al.*, 2010 ▸).
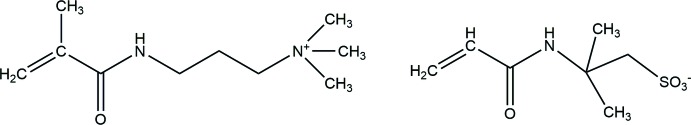


## Structural commentary   

**Figure 1:**
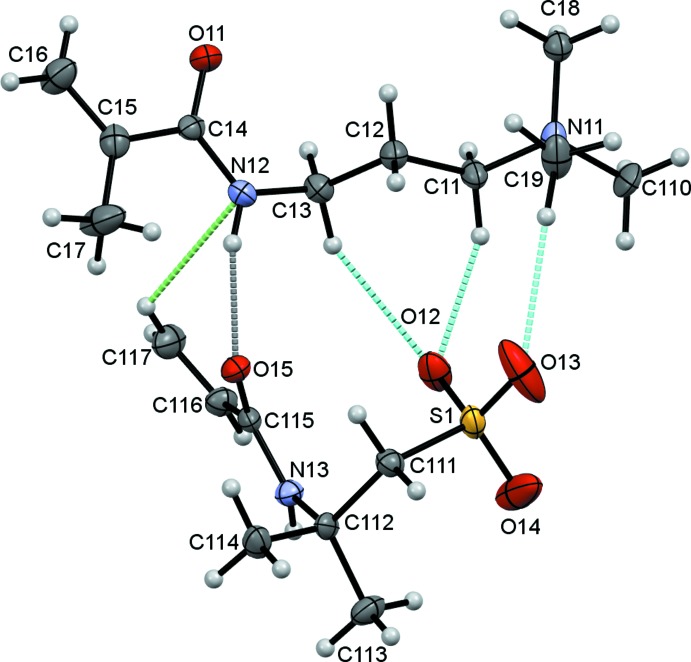
Salt 1 of the title compound showing the atom numbering with ellipsoids drawn at the 50% probability level. N—H⋯O, C—H⋯O and C—H⋯N hydrogen bonds are drawn as dashed grey, cyan and green lines, respectively.

The title compound (1) is a salt consisting of a 3-methacryl­amido-*N*,*N*,*N*-tri­methyl­propan-1-aminium cation and a 2-acryl­amido-2-methyl­propane-1-sulfonate anion. The asymmetric unit contains two unique pairs of cations and anions and the individual cation/anion pairs are shown in Figs. 1 ▸ and 2 ▸. In the numbering scheme the two salts are distinguished by leading 1 and 2 characters. A feature of both cation/anion pairs is the substantial number of inter­molecular contacts, N—H⋯O, C—H⋯O and weaker C—H⋯N hydrogen bonds, Table 1 ▸, linking the cations to the anions, with the O12 and O22 atoms acting as bifurcated acceptors enclosing 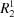
(6) ring motifs in each case.

**Figure 3:**
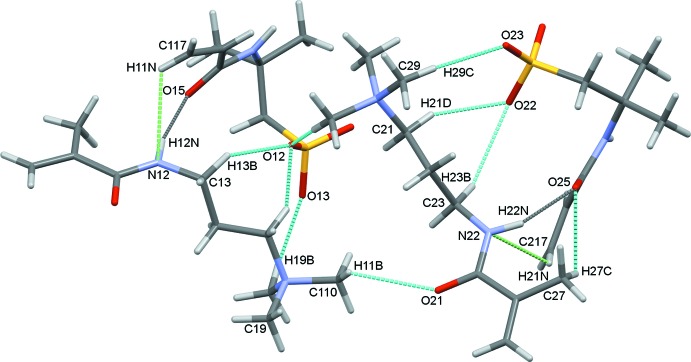
Inter­molecular contacts in the asymmetric unit of (1).

**Figure 4:**
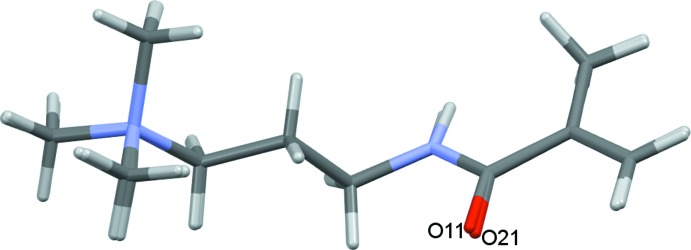
An overlay of the two unique cations of (1), r.m.s. deviation 0.0561 Å.

**Figure 5:**
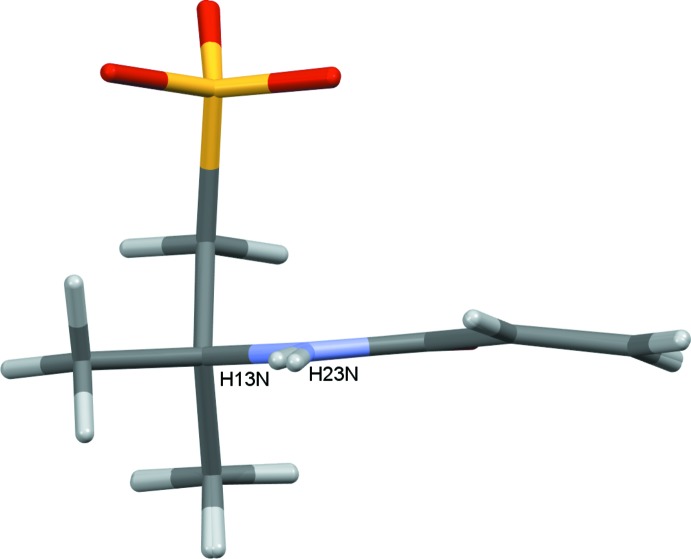
An overlay of the two unique anions of (1), r.m.s. deviation 0.0228 Å.

In the asymmetric unit the cations and anions are inter­connected by further N—H⋯O, C—H⋯O and C—H⋯N hydrogen bonds with O12 and O22 acting as trifurcated and bifurcated acceptors, respectively, Fig. 3 ▸. The unique cation and anions pairs in (1) are reasonably similar to one another. Examination of selected bond distances, Table 2 ▸, confirms this similarity. Furthermore, the individual cations and anions overlay with r.m.s. deviations of only 0.0561 Å for the two cations and 0.0228 Å for the anions (Macrae *et al.*, 2008 ▸). For the cations the most significant variations occur around the amide unit and for one of the methyl groups of the tri­methyl­amine substituent, Fig. 4 ▸. The anions are even more closely comparable with only small variations around the amide N atoms and the vinyl groups, Fig. 5 ▸.

While the cations both adopt stretched arrangements, aided by the central propyl units, the anions are U-shaped with the acryl­amide and sulfonate residues on opposite vertices of the U. The relative conformations of the C=O and vinyl double bonds within the C115 and C215 acryl­amide substituents of the anions are *s-cis*, as found in similar compounds (Goswami *et al.*, 2017 ▸). The two methacryl­amide residues of the cations are similarly arranged.

## Supra­molecular features   

**Figure 6:**
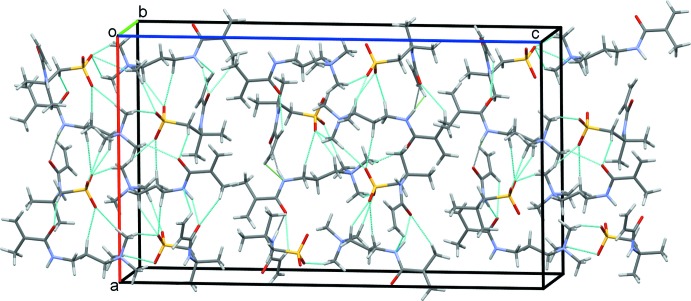
Sheets of the cations and anions of (1) in the *ac* plane. All hydrogen bonds are shown as dashed cyan lines.

**Figure 7:**
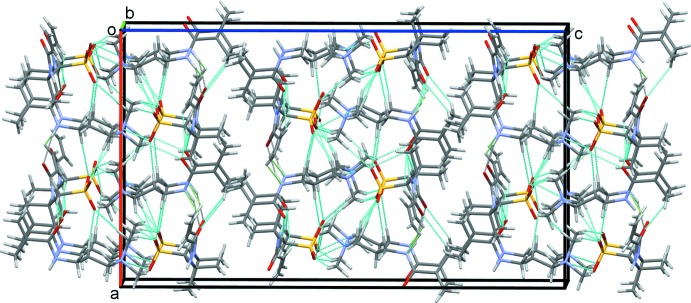
Overall packing of the title compound viewed along the *b-*axis direction.

In the crystal, a series of N—H⋯O and C—H⋯O hydrogen bonds, Table 1 ▸, form double chains of cations and anions along the *a* axis with adjacent double chains forming sheets in the *ac* plane, Fig. 6 ▸. These sheets are stacked along the *b-*axis direction by additional C—H⋯O hydrogen bonds, Fig. 7 ▸.

## Hirshfeld Analysis   

**Figure 8:**
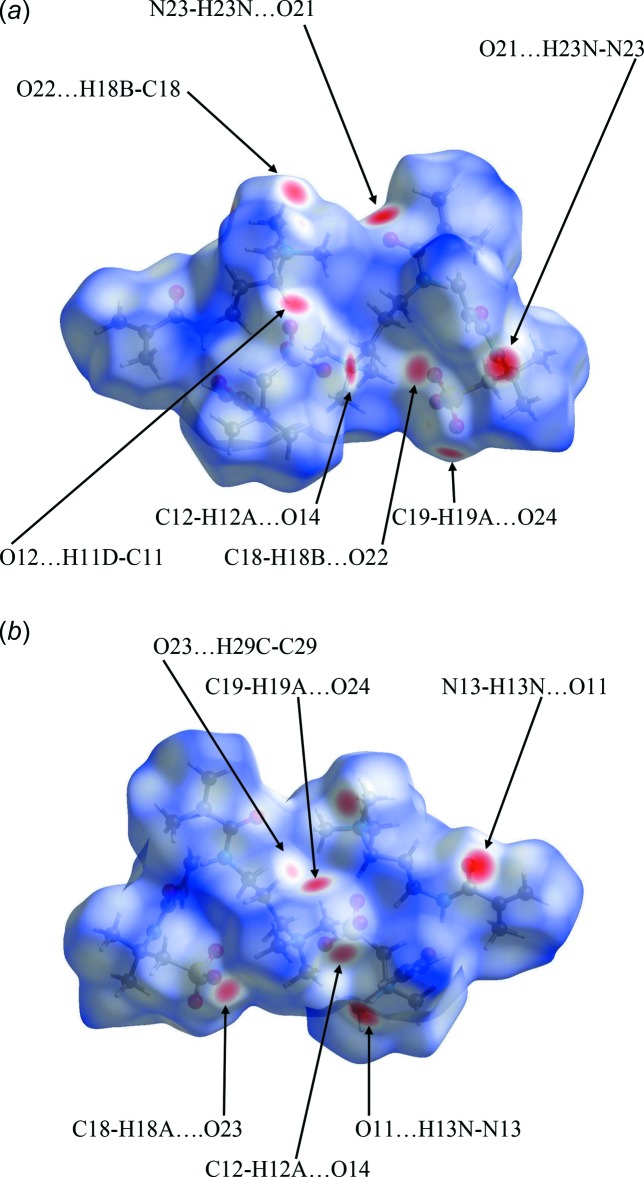
Hirshfeld surfaces for opposite faces of the asymmetric unit of (1) mapped over *d*_norm_ in the range −0.5027 to 1.6303 a.u.

**Figure 9:**
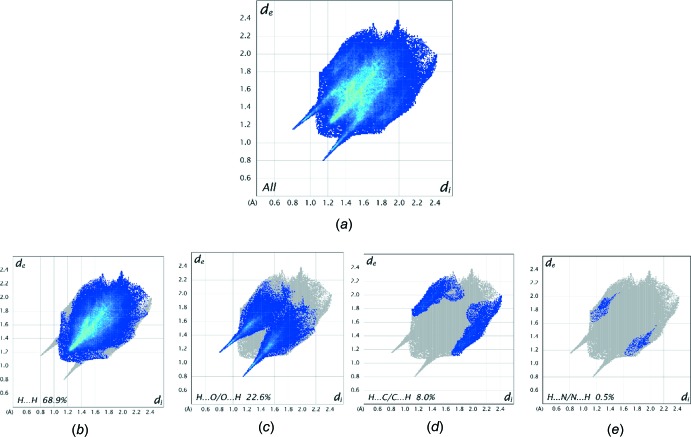
Full two-dimensional fingerprint plots for the asymmetric unit of (1) (*a*) and (*b*)–(*e*) separate contact types for the separate contact types for the asymmetric unit of the salt. These are found to be H⋯H, H⋯O/O⋯H, H⋯C/C⋯H and H⋯N/N⋯H contacts.

Further details of the inter­molecular architecture of this salt are available using Hirshfeld surface analysis (Spackman & Jayatilaka, 2009 ▸) with surfaces and two-dimensional fingerprint plots generated by *CrystalExplorer* (Turner *et al.*, 2017 ▸). Hirshfeld surfaces of the asymmetric unit of the structure which comprises salts 1 and 2, viewed for opposite faces are shown in Fig. 8 ▸(*a*) and 8(*b*). The red circles on the Hirshfeld surfaces correspond to the N—H⋯O and some of the numerous C—H⋯O contacts that play a significant role in stabilizing the packing in this structure. Fingerprint plots of the contacts on the Hirshfeld surface of the asymmetric unit of (1) are shown in Fig. 9 ▸. These comprise H⋯H, H⋯C/C⋯H, and H⋯O/O⋯H and the much weaker and less significant H⋯N/N⋯H contributions. All contacts are detailed in Table 3 ▸.

**Figure 10:**
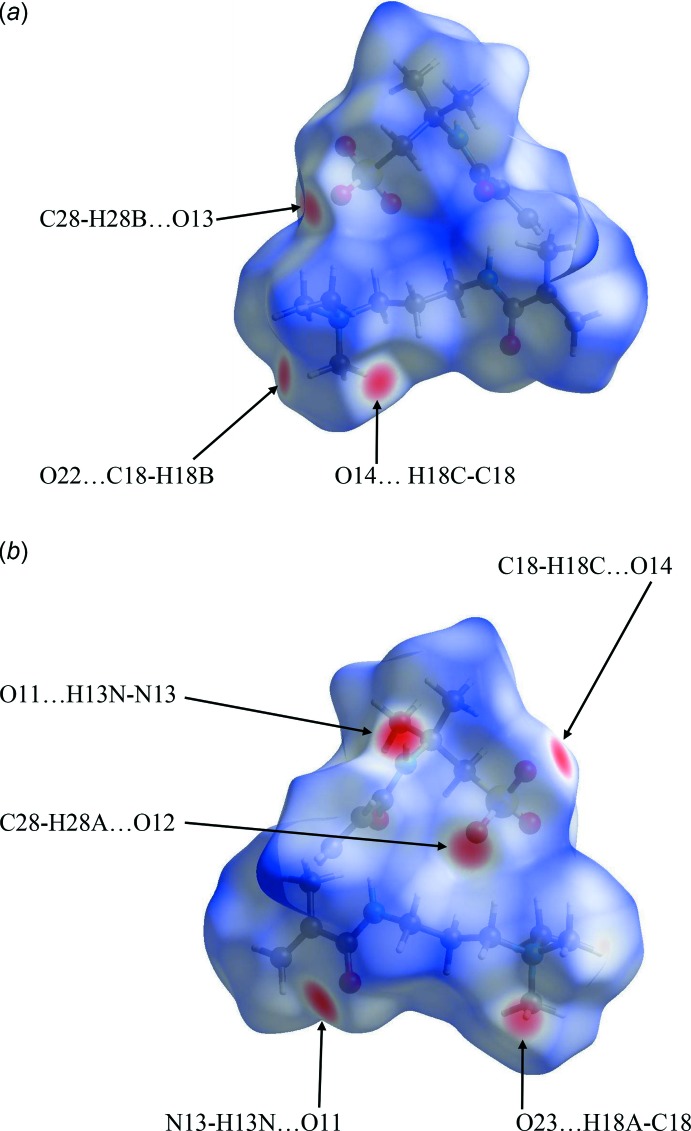
Hirshfeld surfaces for opposite faces of salt 1 mapped over *d*_norm_ in the range −0.4919 to 1.6314 a.u.

**Figure 11:**
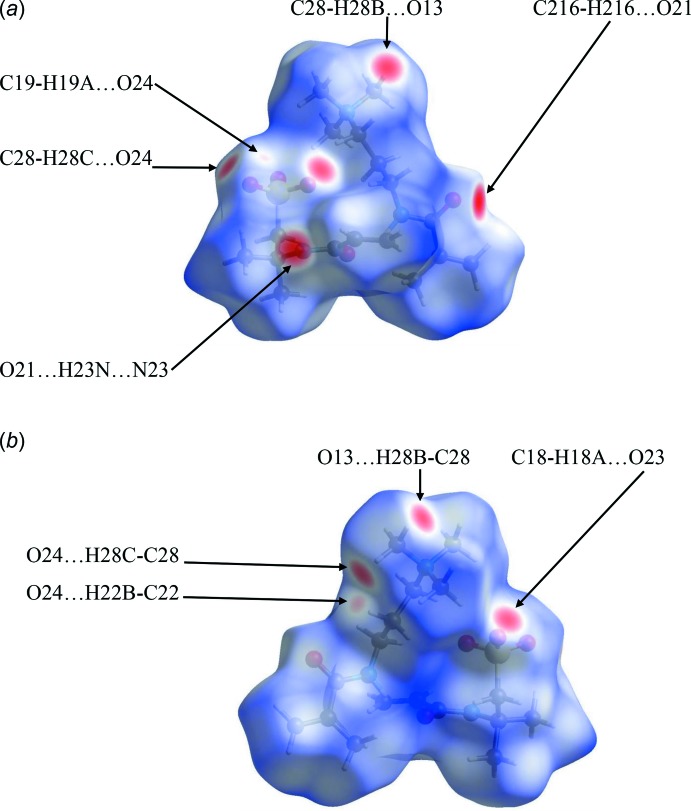
Hirshfeld surfaces for opposite faces of salt 2 mapped over *d*_norm_ in the range −0.5029 to 1.6274 a.u.

The surfaces of the two discrete salt components of the structure can also be examined individually. Fig. 10 ▸(*a*) and 10(*b*) for salt 1 and Fig. 11 ▸(*a*) and 11(*b*) for salt 2 show the Hirshfeld surfaces of the individual salts 1 and 2, for opposite faces in each case. An immediate observation, strongly supported by the surface area data found in the fingerprint plots, *vide infra*, is that the surface contacts in the two discrete salts are reasonably similar to one another. Such similarities are also signalled by the closely comparable metrical data for the two salts and the results of the overlay experiments on the pairs of cations and anions discussed earlier.

**Figure 12:**
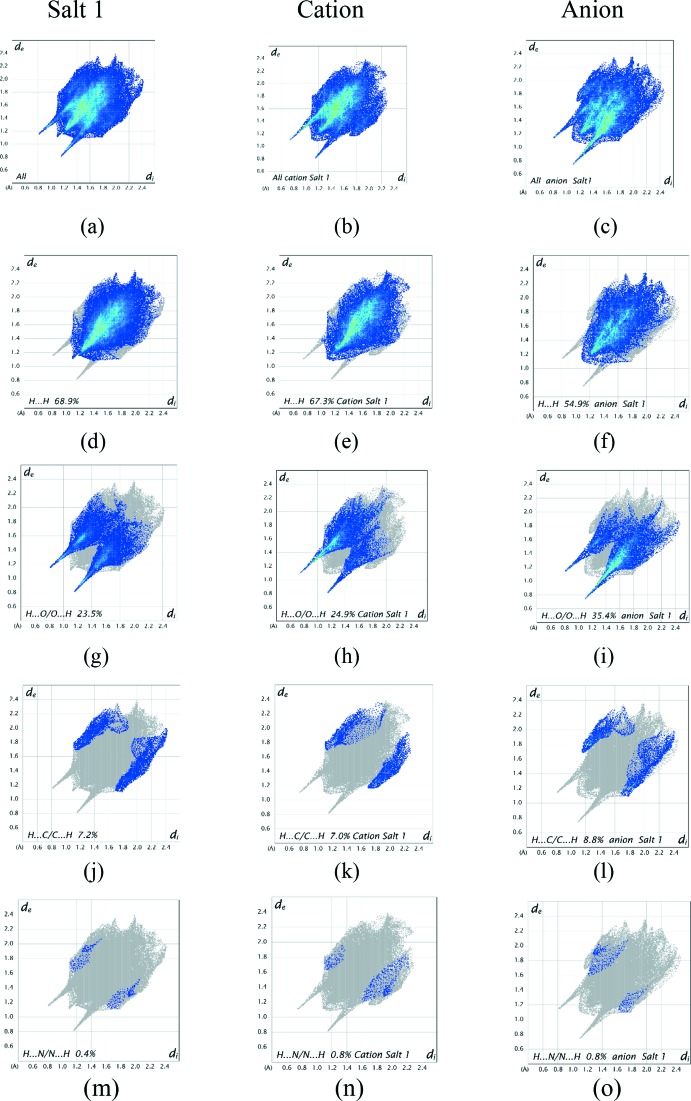
Full two-dimensional fingerprint plots for salt 1 (*a*) its cation (*b*) and anion (*c*); (*d*)–(*o*) separate contact types for the salt, cation and anion systems respectively. These are found to be H⋯H, H⋯O/O⋯H, H⋯C/C⋯H and H⋯N/N⋯H contacts.

**Figure 13:**
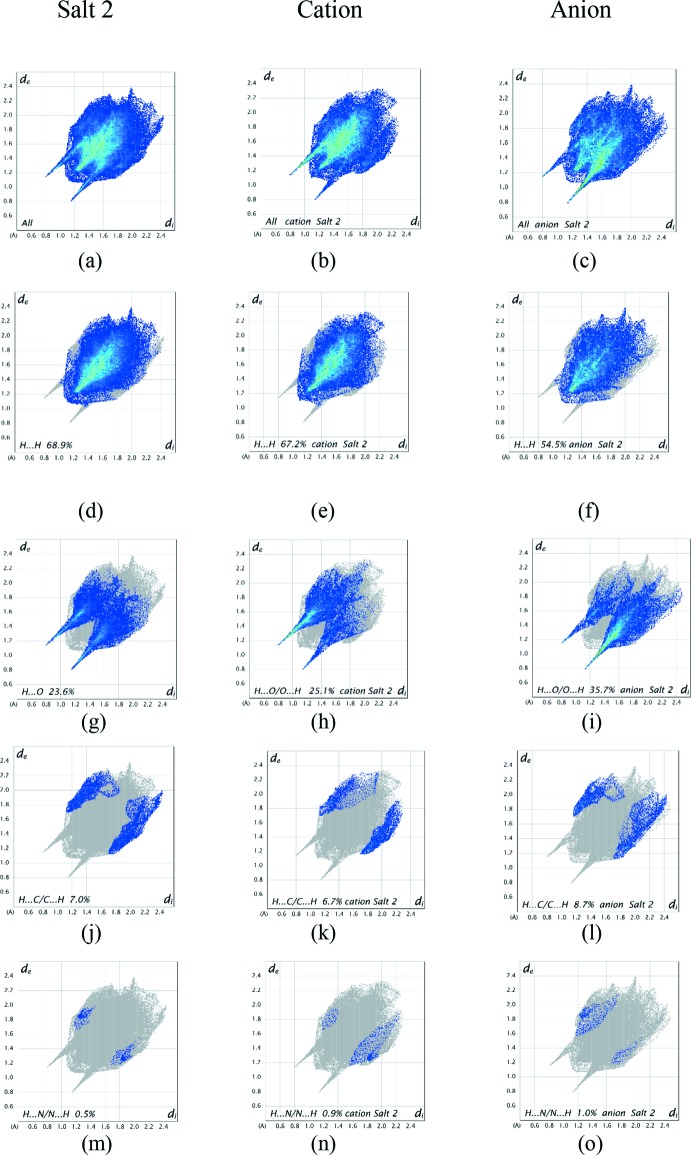
Full two-dimensional fingerprint plots for salt 2 (*a*) its cation (*b*) and anion (*c*); (*d*)–(*o*) separate contact types for the salt, cation and anion systems respectively. These are found to be H⋯H, H⋯O/O⋯H, H⋯C/C⋯H and H⋯N/N⋯H contacts.

It is also instructive to investigate the differences in contacts for the discrete cation and anion components of both salts by recording fingerprint plots for the two salts together with those of the discrete cations and anions. All of the surface contributions for the individual salts and their component cations and anions are shown in Table 4 ▸, with fingerprint plots for these contacts displayed in Fig. 12 ▸ for salt 1 and Fig. 13 ▸ for salt 2. The fingerprint plots for the two salts are closely analogous as indeed are the percentage contribution figures in Table 4 ▸, further highlighting their similarities. The most notable differences between the values for the salt and its components are that the H⋯H van der Waals inter­actions are significantly greater for the cations in comparison to the anions, while the anion shows considerable increases in the H⋯O/O⋯H contacts reflecting the prominent role of the sulfonate O atoms in hydrogen bond formation. The H⋯N/N⋯H contributions to all of the surfaces are very weak but are included for completeness.

## Database survey   

The Cambridge Structural Database (version 5.40 Nov 2018 with update of May 2019; Groom *et al.* 2016 ▸) contains structures of 66 acryl­amide and 41 methacryl­amide derivatives including acryl­amide itself (ARCLAM01; Zhou *et al.* 2007 ▸) and both the *s-cis* (WANSAG) and *s-trans* (WANSAG01) conformations of methacryl­amide (Guo *et al.* 2005 ▸). However, these results show that both components of this salt are unusual with no hits for any structures of related methyl­acryl­amido cations nor acryl­amido­sulfonate anions. Indeed, the only structure showing even a moderately close relationship to either of the mol­ecules reported here is *N*,*N*,*N*′,*N*′-tetra­methyl-*N*′′-[3-(tri­methyl­aza­nium­yl)prop­yl]guanidinium bis­(tetra­phenyl­borate) acetone solvate (Tiritiris, 2013 ▸) that contains the Me_3_N^+^(CH_2_)_3_NH- fragment.

## Synthesis and crystallization   

The title compound was prepared *via* an argentometric mixing approach (Li *et al.*, 2010 ▸) from the silver salt of 2-acryl­amido-2-methyl-1-propane­sulfonic acid (*AMPS*) and 3-(meth­acryl­oyl­amino)­propyl-tri­methyl­ammonium chloride (*MPT* Cl). After filtration of the AgCl precipitate, the solution was freeze-dried and the ion-pair comonomers recrystallized from dioxane.

^1^H NMR (400 MHz, DMSO-*d*_6_): δ 8.36 (*br s*, 1H, *AMPS* amide H), 8.06 (*br s*, 1H, *MPT* amide H), 6.09–5.89 (*m*, 2H, *AMPS* =CH_2_), 5.69 (*m*, 1H, *MPT*=CH), 5.48 (*m*, 1H, *AMPS* =CH), 5.32 (*m*, 1H *MPT*=CH), 3.31–3.22 (*m*, 2H, *MPT* CH_2_), 3.15 (*m*, 2H, *MPT* CH_2_), 3.02 (*s*, 9H, *MPT* CH_3_), 2.72 (*s*, 2H, *AMPS* CH_2_), 1.91–1.79 (*m*, 2H, *MPT* CH_2_), 1.79 (*s*, 3H, *MPT*=CCH_3_), 1.41 (*s*, 6H, *AMPS* CH_3_).

## Refinement   

Crystal data, data collection and structure refinement details are summarized in Table 5 ▸. N—H hydrogen atoms were located in a difference-Fourier map and their coordinates were refined with *U*_iso_(H) = 1.2*U*_eq_(N). All H atoms bound to carbon were refined using a riding model with *d*(C—H) = 0.95 Å and *U*_iso_(H) = 1.2*U*_eq_(C) for aromatic and vinyl H atoms, *d*(C—H) = 0.99 Å and *U*_iso_(H) = 1.2*U*_eq_(C) for methyl­ene and *d*(C—H) = 0.98 Å and *U*_iso_(H) = 1.5*U*_eq_(C) for methyl H atoms. The crystal studied was refined as a two-component inversion twin with a 0.58 (4):0.42 (4) domain ratio. Two reflections with *F*_o_ >>> *F*_c_ were omitted from the final refinement cycles.

## Supplementary Material

Crystal structure: contains datablock(s) I, global. DOI: 10.1107/S2056989019012003/vm2221sup1.cif

Structure factors: contains datablock(s) I. DOI: 10.1107/S2056989019012003/vm2221Isup2.hkl

Supporting information file. DOI: 10.1107/S2056989019012003/vm2221Isup3.cml

CCDC reference: 1950279

Additional supporting information:  crystallographic information; 3D view; checkCIF report

## Figures and Tables

**Figure 2:**
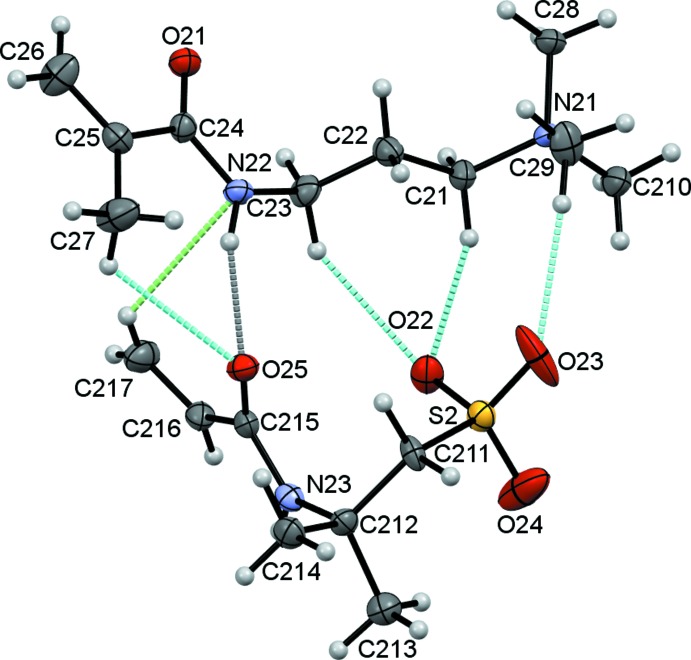
Salt 2 of (1) showing the atom numbering with ellipsoids drawn at the 50% probability level.

**Table 1:** Hydrogen-bond geometry (Å, °)

*D*—H⋯*A*	*D*—H	H⋯*A*	*D*⋯*A*	*D*—H⋯*A*
N12—H12*N*⋯O15	0.84 (7)	2.02 (7)	2.841 (6)	167 (7)
N13—H13*N*⋯O11^i^	0.88 (7)	2.10 (7)	2.943 (6)	162 (6)
N22—H22*N*⋯O25	0.93 (7)	2.00 (7)	2.865 (6)	154 (6)
N23—H23*N*⋯O21^ii^	0.82 (7)	2.15 (7)	2.961 (6)	174 (7)
C11—H11*D*⋯O12	0.99	2.31	3.216 (8)	151
C12—H12*A*⋯O14^iii^	0.99	2.68	3.583 (8)	151
C13—H13*B*⋯O12	0.99	2.69	3.463 (8)	135
C18—H18*C*⋯O14^iii^	0.98	2.23	3.182 (10)	164
C18—H18*B*⋯O22^iii^	0.98	2.25	3.192 (8)	160
C18—H18*A*⋯O23^iv^	0.98	2.28	3.226 (9)	162
C19—H19*A*⋯O24^iii^	0.98	2.63	3.555 (10)	157
C110—H11*B*⋯O21	0.98	2.65	3.182 (11)	114
C116—H116⋯O11^i^	0.95	2.68	3.375 (7)	131
C117—H11*N*⋯N12	0.95	2.73	3.338 (8)	123
C21—H21*D*⋯O22	0.99	2.34	3.236 (8)	151
C22—H22*B*⋯O24^iv^	0.99	2.53	3.463 (8)	157
C23—H23*B*⋯O22	0.99	2.65	3.442 (7)	137
C28—H28*A*⋯O12	0.98	2.20	3.162 (9)	166
C28—H28*B*⋯O13^v^	0.98	2.27	3.195 (9)	158
C29—H29*C*⋯O23	0.98	2.41	3.377 (10)	169
C211—H21*F*⋯O21^i^	0.99	2.71	3.674 (8)	164
C216—H216⋯O21^ii^	0.95	2.69	3.405 (7)	132

Symmetry codes: (i) 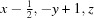; (ii) 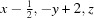; (iii) 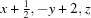; (iv) 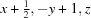; (v) 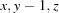.

**Table 2:** Selected bond lengths (Å) for salts 1 and 2

Salt 1		Salt 2	
C18—N11	1.479 (10)	C28—N21	1.504 (9)
C19—N11	1.506 (9)	C29—N21	1.506 (9)
C110—N11	1.498 (11)	C210—N21	1.504 (10)
N11—C11	1.511 (8)	N21—C21	1.500 (8)
C13—N12	1.463 (7)	C23—N22	1.457 (7)
N12—C14	1.330 (7)	N22—C24	1.338 (7)
C14—O11	1.239 (7)	C24—O21	1.236 (7)
C15—C16	1.367 (9)	C25—C26	1.352 (9)
O12—S1	1.434 (5)	O22—S2	1.436 (4)
O13—S1	1.447 (6)	O23—S2	1.437 (6)
O14—S1	1.436 (7)	O24—S2	1.432 (7)
S1—C111	1.778 (8)	S2—C211	1.786 (8)
N13—C115	1.333 (7)	N23—C215	1.338 (7)
C115—O15	1.245 (7)	C215—O25	1.235 (7)
C116—C117	1.304 (9)	C216—C217	1.323 (9)

**Table 3:** Percentage contributions of the inter­atomic contacts to the Hirshfeld surface of the asymmetric unit of (1)

Contacts	Included surface area (%)
H⋯H	68.9
H⋯O/O⋯H	22.6
H⋯C/C⋯H	8.0
H⋯N/N⋯H	0.5

**Table 4:** Percentage contributions of the inter­atomic contacts to the Hirshfeld surface of the individual salts of (1)

Contact	Salt 1	Cation	Anion	Salt 2	Cation	Anion
H⋯H	68.9	67.3	54.9	68.9	67.2	54.5
H⋯O/O⋯H	23.5	24.9	35.4	23.6	25.1	35.7
H⋯C/C⋯H	7.2	7.0	8.8	7.0	6.7	8.7
H⋯N/N⋯H	0.4	0.8	0.8	0.5	0.9	1.0

**Table 5:** Experimental details

Crystal data	
Chemical formula	C_10_H_21_N_2_O^+^·C_7_H_12_NO_4_S^−^
*M*_r_	391.52
Crystal system, space group	Orthorhombic, *P* *c* *a*2_1_
Temperature (K)	100
*a*, *b*, *c* (Å)	17.5093 (7), 7.8052 (3), 30.3155 (13)
*V* (Å^3^)	4143.0 (3)
*Z*	8
Radiation type	Cu *K*α
μ (mm^−1^)	1.65
Crystal size (mm)	0.46 × 0.27 × 0.11

Data collection	
Diffractometer	Rigaku Oxford Diffraction SuperNova, Dual, Cu at zero, Atlas
Absorption correction	Multi-scan (*CrysAlis PRO*; Rigaku OD, 2018 ▸)
*T*_min_, *T*_max_	0.589, 1.000
No. of measured, independent and observed [*I* > 2σ(*I*)] reflections	10436, 5961, 5040
*R*_int_	0.054
(sin θ/λ)_max_ (Å^−1^)	0.620

Refinement	
*R*[*F* ^2^ > 2σ(*F* ^2^)], *wR*(*F* ^2^), *S*	0.060, 0.173, 1.03
No. of reflections	5961
No. of parameters	494
No. of restraints	31
H-atom treatment	H atoms treated by a mixture of independent and constrained refinement
Δρ_max_, Δρ_min_ (e Å^−3^)	0.64, −0.32
Absolute structure	Refined as an inversion twin.
Absolute structure parameter	0.47 (4)

Computer programs: *CrysAlis PRO* (Rigaku OD, 2018 ▸), *SHELXT* (Sheldrick, 2015*a* ▸), *SHELXL2018* (Sheldrick, 2015*b* ▸), *TITAN* (Hunter & Simpson, 1999 ▸), *Mercury* (Macrae *et al.*, 2008 ▸), *enCIFer* (Allen *et al.*, 2004 ▸), *PLATON* (Spek, 2009 ▸), *publCIF* (Westrip 2010 ▸) and *WinGX* (Farrugia, 2012 ▸).

## supplementary crystallographic information

### Crystal data

**Table:**

C_10_H_21_N_2_O^+^·C_7_H_12_NO_4_S^−^	*D*_x_ = 1.255 Mg m^−^^3^
*M**_r_* = 391.52	Cu *K*α radiation, λ = 1.54184 Å
Orthorhombic, *P**c**a*2_1_	Cell parameters from 4725 reflections
*a* = 17.5093 (7) Å	θ = 5.2–72.8°
*b* = 7.8052 (3) Å	µ = 1.65 mm^−^^1^
*c* = 30.3155 (13) Å	*T* = 100 K
*V* = 4143.0 (3) Å^3^	Plate, colourless
*Z* = 8	0.46 × 0.27 × 0.11 mm
*F*(000) = 1696	

### Data collection

**Table:**

Rigaku Oxford Diffraction SuperNova, Dual, Cu at zero, Atlas diffractometer	5961 independent reflections
Radiation source: SuperNova (Cu) X-ray Source	5040 reflections with *I* > 2σ(*I*)
Detector resolution: 5.1725 pixels mm^-1^	*R*_int_ = 0.054
ω scans	θ_max_ = 72.8°, θ_min_ = 5.1°
Absorption correction: multi-scan (CrysAlis PRO; Rigaku OD, 2018)	*h* = −21→15
*T*_min_ = 0.589, *T*_max_ = 1.000	*k* = −9→6
10436 measured reflections	*l* = −36→36

### Refinement

**Table:**

Refinement on *F*^2^	Hydrogen site location: mixed
Least-squares matrix: full	H atoms treated by a mixture of independent and constrained refinement
*R*[*F*^2^ > 2σ(*F*^2^)] = 0.060	*w* = 1/[σ^2^(*F*_o_^2^) + (0.1018*P*)^2^ + 1.0621*P*] where *P* = (*F*_o_^2^ + 2*F*_c_^2^)/3
*wR*(*F*^2^) = 0.173	(Δ/σ)_max_ < 0.001
*S* = 1.03	Δρ_max_ = 0.64 e Å^−^^3^
5961 reflections	Δρ_min_ = −0.32 e Å^−^^3^
494 parameters	Absolute structure: Refined as an inversion twin.
31 restraints	Absolute structure parameter: 0.47 (4)

### Special details

**Table:**

Geometry. All esds (except the esd in the dihedral angle between two l.s. planes) are estimated using the full covariance matrix. The cell esds are taken into account individually in the estimation of esds in distances, angles and torsion angles; correlations between esds in cell parameters are only used when they are defined by crystal symmetry. An approximate (isotropic) treatment of cell esds is used for estimating esds involving l.s. planes.
Refinement. Refined as a 2-component inversion twin. Two reflections with Fo >>> Fc were omitted from the final refinement cycles.

### Fractional atomic coordinates and isotropic or equivalent isotropic displacement parameters (Å^2^)

**Table:**

	*x*	*y*	*z*	*U*_iso_*/*U*_eq_	
C18	0.6830 (4)	0.9831 (8)	0.5183 (3)	0.0247 (16)	
H18A	0.690198	0.878168	0.535604	0.037*	
H18B	0.693085	1.083025	0.537028	0.037*	
H18C	0.718325	0.983217	0.493281	0.037*	
C19	0.5921 (5)	1.1545 (9)	0.4766 (3)	0.0349 (17)	
H19A	0.595183	1.252064	0.496889	0.052*	
H19B	0.541887	1.153142	0.462385	0.052*	
H19C	0.631985	1.165139	0.454063	0.052*	
C110	0.5495 (5)	0.9882 (10)	0.5403 (3)	0.037 (2)	
H11A	0.560070	1.086234	0.559514	0.055*	
H11B	0.556169	0.881640	0.556894	0.055*	
H11C	0.496835	0.995378	0.529420	0.055*	
N11	0.6035 (4)	0.9905 (6)	0.5019 (2)	0.0258 (14)	
C11	0.5863 (4)	0.8365 (8)	0.4734 (2)	0.0247 (14)	
H11D	0.530261	0.826601	0.470045	0.030*	
H11E	0.604392	0.732378	0.488861	0.030*	
C12	0.6220 (3)	0.8411 (7)	0.4280 (2)	0.0250 (12)	
H12A	0.677882	0.857686	0.430586	0.030*	
H12B	0.600668	0.937774	0.410867	0.030*	
C13	0.6053 (3)	0.6730 (7)	0.4046 (2)	0.0249 (12)	
H13A	0.637844	0.581781	0.417331	0.030*	
H13B	0.551289	0.640957	0.409807	0.030*	
N12	0.6191 (3)	0.6838 (6)	0.35712 (17)	0.0224 (10)	
H12N	0.578 (4)	0.693 (9)	0.343 (3)	0.027*	
C14	0.6881 (3)	0.6571 (7)	0.3401 (2)	0.0198 (11)	
O11	0.7443 (2)	0.6223 (6)	0.36331 (15)	0.0272 (9)	
C15	0.6946 (4)	0.6717 (8)	0.2908 (2)	0.0261 (13)	
C16	0.7639 (4)	0.6354 (9)	0.2722 (2)	0.0358 (15)	
H16A	0.770611	0.644668	0.241236	0.043*	
H16B	0.805393	0.600812	0.290379	0.043*	
C17	0.6299 (4)	0.7244 (13)	0.2650 (3)	0.047 (2)	
H17A	0.644894	0.733716	0.233933	0.071*	
H17B	0.611814	0.835987	0.275506	0.071*	
H17C	0.588996	0.639623	0.267955	0.071*	
O12	0.4219 (3)	0.8164 (6)	0.42637 (18)	0.0348 (11)	
O13	0.4113 (5)	1.1199 (7)	0.4319 (2)	0.067 (2)	
O14	0.3005 (3)	0.9400 (12)	0.4408 (2)	0.070 (2)	
S1	0.37335 (9)	0.9620 (2)	0.41979 (5)	0.0265 (4)	
C111	0.3578 (4)	0.9838 (7)	0.3621 (3)	0.0221 (15)	
H11F	0.330598	1.093499	0.357282	0.026*	
H11G	0.408354	0.994452	0.347790	0.026*	
C112	0.3129 (3)	0.8423 (7)	0.33741 (19)	0.0195 (11)	
C113	0.2292 (3)	0.8345 (8)	0.3513 (2)	0.0302 (14)	
H11H	0.203429	0.742638	0.335013	0.045*	
H11I	0.204478	0.944182	0.344692	0.045*	
H11J	0.225960	0.811578	0.382995	0.045*	
C114	0.3169 (4)	0.8832 (9)	0.2880 (2)	0.0285 (13)	
H11K	0.370443	0.891877	0.278843	0.043*	
H11L	0.290935	0.992159	0.282197	0.043*	
H11M	0.291816	0.791602	0.271238	0.043*	
N13	0.3453 (3)	0.6713 (6)	0.34566 (17)	0.0204 (10)	
H13N	0.309 (4)	0.600 (9)	0.354 (2)	0.025*	
C115	0.4176 (3)	0.6236 (7)	0.33943 (19)	0.0198 (11)	
O15	0.4681 (2)	0.7184 (5)	0.32397 (14)	0.0233 (8)	
C116	0.4340 (3)	0.4425 (8)	0.3526 (2)	0.0238 (12)	
H116	0.396813	0.383181	0.369559	0.029*	
C117	0.4965 (4)	0.3618 (8)	0.3420 (3)	0.0337 (15)	
H11N	0.534618	0.418056	0.324986	0.040*	
H11O	0.504003	0.246658	0.351177	0.040*	
C28	0.4323 (4)	0.4759 (9)	0.4825 (3)	0.0251 (16)	
H28A	0.438522	0.577869	0.464020	0.038*	
H28B	0.440159	0.372881	0.464636	0.038*	
H28C	0.469854	0.478512	0.506503	0.038*	
C29	0.3427 (5)	0.3137 (9)	0.5288 (3)	0.0353 (17)	
H29A	0.381784	0.309600	0.551935	0.053*	
H29B	0.347640	0.212808	0.509783	0.053*	
H29C	0.291909	0.314495	0.542420	0.053*	
C210	0.2971 (4)	0.4666 (10)	0.4641 (3)	0.0319 (16)	
H21A	0.245390	0.450262	0.475731	0.048*	
H21B	0.310127	0.370769	0.444590	0.048*	
H21C	0.299313	0.574064	0.447434	0.048*	
N21	0.3530 (3)	0.4736 (6)	0.5017 (2)	0.0192 (11)	
C21	0.3369 (4)	0.6330 (8)	0.5277 (2)	0.0246 (14)	
H21D	0.280949	0.644582	0.531300	0.030*	
H21E	0.355032	0.733184	0.510638	0.030*	
C22	0.3744 (3)	0.6368 (8)	0.5736 (2)	0.0264 (12)	
H22A	0.353621	0.543142	0.592074	0.032*	
H22B	0.430212	0.620149	0.570793	0.032*	
C23	0.3579 (3)	0.8094 (8)	0.59519 (19)	0.0257 (13)	
H23A	0.390392	0.898458	0.581496	0.031*	
H23B	0.303840	0.840767	0.589997	0.031*	
N22	0.3725 (3)	0.8040 (6)	0.64248 (17)	0.0223 (10)	
H22N	0.332 (4)	0.785 (9)	0.662 (2)	0.027*	
C24	0.4405 (3)	0.8420 (7)	0.6600 (2)	0.0214 (11)	
O21	0.4954 (2)	0.8854 (5)	0.63690 (15)	0.0255 (9)	
C25	0.4467 (3)	0.8314 (8)	0.7094 (2)	0.0242 (12)	
C26	0.5143 (4)	0.8731 (9)	0.7281 (2)	0.0354 (15)	
H26A	0.520137	0.868026	0.759239	0.042*	
H26B	0.555964	0.907559	0.710104	0.042*	
C27	0.3810 (4)	0.7778 (11)	0.7356 (2)	0.0415 (17)	
H27A	0.395103	0.775397	0.766843	0.062*	
H27B	0.364896	0.663109	0.726266	0.062*	
H27C	0.338970	0.858807	0.731144	0.062*	
O22	0.1734 (3)	0.6760 (6)	0.57622 (17)	0.0310 (10)	
O23	0.1667 (4)	0.3726 (7)	0.5717 (2)	0.0591 (18)	
O24	0.0536 (3)	0.5449 (11)	0.5619 (2)	0.0624 (19)	
S2	0.12650 (9)	0.52727 (18)	0.58283 (5)	0.0240 (4)	
C211	0.1106 (5)	0.5078 (7)	0.6408 (3)	0.0217 (15)	
H21F	0.083063	0.398716	0.645875	0.026*	
H21G	0.161104	0.496867	0.655210	0.026*	
C212	0.0661 (3)	0.6508 (7)	0.6651 (2)	0.0215 (11)	
C213	−0.0178 (3)	0.6588 (8)	0.6509 (2)	0.0304 (14)	
H21H	−0.043509	0.751535	0.666856	0.046*	
H21I	−0.042740	0.549617	0.657698	0.046*	
H21J	−0.020728	0.680539	0.619112	0.046*	
C214	0.0700 (4)	0.6112 (8)	0.7144 (2)	0.0274 (13)	
H21K	0.123502	0.609082	0.723913	0.041*	
H21L	0.046610	0.499244	0.720077	0.041*	
H21M	0.042408	0.699737	0.730856	0.041*	
N23	0.0982 (3)	0.8228 (6)	0.65627 (16)	0.0205 (10)	
H23N	0.071 (4)	0.904 (10)	0.649 (2)	0.025*	
C215	0.1711 (3)	0.8691 (8)	0.6618 (2)	0.0215 (12)	
O25	0.2210 (2)	0.7750 (5)	0.67734 (14)	0.0240 (9)	
C216	0.1867 (3)	1.0492 (8)	0.6480 (2)	0.0234 (12)	
H216	0.148817	1.107951	0.631384	0.028*	
C217	0.2507 (3)	1.1311 (8)	0.6577 (3)	0.0346 (16)	
H21N	0.289415	1.075050	0.674228	0.041*	
H21O	0.257890	1.245841	0.648134	0.041*	

### Atomic displacement parameters (Å^2^)

**Table:**

	*U*^11^	*U*^22^	*U*^33^	*U*^12^	*U*^13^	*U*^23^
C18	0.025 (4)	0.027 (3)	0.022 (4)	−0.002 (2)	0.001 (3)	−0.002 (2)
C19	0.054 (5)	0.028 (3)	0.022 (4)	0.017 (3)	−0.003 (3)	0.001 (3)
C110	0.026 (4)	0.055 (5)	0.028 (4)	−0.002 (3)	0.013 (3)	−0.010 (3)
N11	0.031 (3)	0.027 (3)	0.019 (3)	0.003 (2)	0.004 (3)	−0.0038 (19)
C11	0.024 (3)	0.031 (3)	0.019 (3)	−0.008 (2)	0.003 (3)	−0.005 (2)
C12	0.023 (3)	0.030 (3)	0.022 (3)	−0.001 (2)	0.001 (2)	−0.005 (2)
C13	0.024 (3)	0.026 (3)	0.025 (3)	−0.002 (2)	−0.001 (2)	−0.004 (2)
N12	0.020 (2)	0.027 (2)	0.021 (2)	0.0001 (19)	−0.004 (2)	−0.0041 (19)
C14	0.017 (2)	0.015 (2)	0.027 (3)	−0.002 (2)	0.000 (2)	−0.004 (2)
O11	0.0191 (19)	0.035 (2)	0.028 (2)	0.0022 (16)	−0.0012 (17)	−0.0033 (19)
C15	0.031 (3)	0.023 (3)	0.024 (3)	−0.004 (2)	0.004 (2)	−0.001 (2)
C16	0.031 (3)	0.047 (4)	0.029 (3)	0.012 (3)	0.005 (3)	0.009 (3)
C17	0.023 (3)	0.087 (6)	0.032 (4)	0.009 (4)	0.003 (3)	0.018 (4)
O12	0.046 (2)	0.030 (2)	0.028 (3)	0.0111 (19)	−0.012 (2)	−0.002 (2)
O13	0.128 (6)	0.029 (3)	0.044 (4)	−0.008 (3)	−0.038 (4)	−0.004 (3)
O14	0.031 (3)	0.154 (6)	0.024 (3)	0.020 (4)	0.002 (2)	0.006 (4)
S1	0.0313 (9)	0.0304 (8)	0.0177 (8)	0.0080 (6)	−0.0038 (7)	−0.0061 (7)
C111	0.021 (3)	0.021 (3)	0.024 (5)	−0.002 (2)	−0.002 (3)	−0.002 (2)
C112	0.019 (3)	0.015 (2)	0.025 (3)	0.003 (2)	−0.002 (2)	−0.007 (2)
C113	0.020 (3)	0.028 (3)	0.042 (4)	0.005 (2)	0.005 (3)	−0.003 (3)
C114	0.031 (3)	0.032 (3)	0.022 (3)	0.009 (3)	−0.004 (3)	−0.004 (2)
N13	0.017 (2)	0.021 (2)	0.023 (2)	−0.0006 (18)	0.0009 (19)	−0.0006 (19)
C115	0.019 (3)	0.024 (3)	0.016 (3)	−0.003 (2)	0.000 (2)	−0.006 (2)
O15	0.0176 (18)	0.029 (2)	0.024 (2)	−0.0012 (16)	0.0026 (16)	−0.0008 (17)
C116	0.022 (3)	0.021 (3)	0.029 (3)	0.003 (2)	−0.002 (2)	0.001 (3)
C117	0.029 (3)	0.023 (3)	0.049 (4)	0.000 (2)	0.002 (3)	−0.012 (3)
C28	0.017 (3)	0.035 (3)	0.024 (4)	0.000 (2)	0.003 (3)	−0.005 (3)
C29	0.051 (5)	0.024 (3)	0.031 (4)	−0.009 (3)	0.006 (3)	0.002 (3)
C210	0.022 (4)	0.049 (4)	0.025 (4)	0.000 (3)	−0.002 (3)	−0.011 (3)
N21	0.013 (2)	0.025 (2)	0.020 (3)	−0.0014 (18)	0.000 (2)	−0.003 (2)
C21	0.023 (3)	0.029 (3)	0.022 (3)	0.002 (2)	−0.003 (3)	−0.007 (3)
C22	0.025 (3)	0.034 (3)	0.021 (3)	0.005 (2)	0.000 (2)	−0.004 (2)
C23	0.024 (3)	0.034 (3)	0.019 (3)	−0.001 (2)	−0.005 (2)	0.001 (2)
N22	0.016 (2)	0.031 (2)	0.020 (2)	−0.0014 (19)	0.0027 (19)	−0.002 (2)
C24	0.023 (3)	0.020 (3)	0.021 (3)	−0.003 (2)	0.000 (2)	−0.002 (2)
O21	0.0214 (19)	0.030 (2)	0.025 (2)	−0.0029 (17)	0.0025 (17)	−0.0014 (18)
C25	0.026 (3)	0.024 (3)	0.023 (3)	0.002 (2)	0.003 (2)	−0.002 (2)
C26	0.038 (3)	0.043 (4)	0.026 (3)	−0.012 (3)	−0.006 (3)	0.012 (3)
C27	0.028 (3)	0.072 (5)	0.024 (3)	−0.007 (3)	0.000 (3)	0.007 (3)
O22	0.038 (2)	0.031 (2)	0.024 (2)	−0.0106 (18)	0.0061 (19)	−0.0002 (19)
O23	0.116 (5)	0.030 (3)	0.032 (3)	0.013 (3)	0.034 (3)	−0.006 (2)
O24	0.034 (3)	0.130 (5)	0.023 (3)	−0.025 (3)	−0.003 (2)	0.006 (4)
S2	0.0250 (8)	0.0296 (8)	0.0173 (8)	−0.0039 (6)	0.0017 (6)	−0.0010 (7)
C211	0.034 (4)	0.017 (3)	0.014 (4)	0.005 (2)	0.000 (3)	−0.0022 (19)
C212	0.019 (2)	0.020 (3)	0.026 (3)	−0.004 (2)	0.003 (2)	−0.002 (2)
C213	0.026 (3)	0.032 (3)	0.033 (4)	0.000 (2)	−0.001 (3)	−0.002 (3)
C214	0.028 (3)	0.033 (3)	0.022 (3)	−0.004 (2)	0.005 (2)	−0.003 (3)
N23	0.022 (2)	0.019 (2)	0.021 (2)	0.0039 (18)	−0.0001 (19)	0.0009 (19)
C215	0.020 (3)	0.025 (3)	0.019 (3)	0.000 (2)	0.001 (2)	−0.007 (2)
O25	0.0188 (19)	0.029 (2)	0.024 (2)	0.0009 (16)	−0.0004 (16)	0.0006 (18)
C216	0.022 (3)	0.023 (3)	0.025 (3)	0.004 (2)	−0.003 (2)	−0.003 (3)
C217	0.025 (3)	0.025 (3)	0.053 (4)	−0.003 (2)	0.001 (3)	−0.004 (3)

### Geometric parameters (Å, º)

**Table:**

C18—N11	1.479 (10)	C28—N21	1.504 (9)
C18—H18A	0.9800	C28—H28A	0.9800
C18—H18B	0.9800	C28—H28B	0.9800
C18—H18C	0.9800	C28—H28C	0.9800
C19—N11	1.506 (9)	C29—N21	1.506 (9)
C19—H19A	0.9800	C29—H29A	0.9800
C19—H19B	0.9800	C29—H29B	0.9800
C19—H19C	0.9800	C29—H29C	0.9800
C110—N11	1.498 (11)	C210—N21	1.504 (10)
C110—H11A	0.9800	C210—H21A	0.9800
C110—H11B	0.9800	C210—H21B	0.9800
C110—H11C	0.9800	C210—H21C	0.9800
N11—C11	1.511 (8)	N21—C21	1.500 (8)
C11—C12	1.511 (9)	C21—C22	1.539 (9)
C11—H11D	0.9900	C21—H21D	0.9900
C11—H11E	0.9900	C21—H21E	0.9900
C12—C13	1.520 (8)	C22—C23	1.525 (8)
C12—H12A	0.9900	C22—H22A	0.9900
C12—H12B	0.9900	C22—H22B	0.9900
C13—N12	1.463 (7)	C23—N22	1.457 (7)
C13—H13A	0.9900	C23—H23A	0.9900
C13—H13B	0.9900	C23—H23B	0.9900
N12—C14	1.330 (7)	N22—C24	1.338 (7)
N12—H12N	0.84 (7)	N22—H22N	0.93 (7)
C14—O11	1.239 (7)	C24—O21	1.236 (7)
C14—C15	1.505 (8)	C24—C25	1.505 (8)
C15—C16	1.367 (9)	C25—C26	1.352 (9)
C15—C17	1.436 (9)	C25—C27	1.457 (9)
C16—H16A	0.9500	C26—H26A	0.9500
C16—H16B	0.9500	C26—H26B	0.9500
C17—H17A	0.9800	C27—H27A	0.9800
C17—H17B	0.9800	C27—H27B	0.9800
C17—H17C	0.9800	C27—H27C	0.9800
O12—S1	1.434 (5)	O22—S2	1.436 (4)
O13—S1	1.447 (6)	O23—S2	1.437 (6)
O14—S1	1.436 (7)	O24—S2	1.432 (7)
S1—C111	1.778 (8)	S2—C211	1.786 (8)
C111—C112	1.548 (8)	C211—C212	1.547 (8)
C111—H11F	0.9900	C211—H21F	0.9900
C111—H11G	0.9900	C211—H21G	0.9900
C112—N13	1.472 (7)	C212—N23	1.479 (7)
C112—C113	1.526 (7)	C212—C214	1.529 (8)
C112—C114	1.534 (8)	C212—C213	1.532 (8)
C113—H11H	0.9800	C213—H21H	0.9800
C113—H11I	0.9800	C213—H21I	0.9800
C113—H11J	0.9800	C213—H21J	0.9800
C114—H11K	0.9800	C214—H21K	0.9800
C114—H11L	0.9800	C214—H21L	0.9800
C114—H11M	0.9800	C214—H21M	0.9800
N13—C115	1.333 (7)	N23—C215	1.338 (7)
N13—H13N	0.88 (7)	N23—H23N	0.82 (7)
C115—O15	1.245 (7)	C215—O25	1.235 (7)
C115—C116	1.496 (8)	C215—C216	1.492 (9)
C116—C117	1.304 (9)	C216—C217	1.323 (9)
C116—H116	0.9500	C216—H216	0.9500
C117—H11N	0.9500	C217—H21N	0.9500
C117—H11O	0.9500	C217—H21O	0.9500
			
N11—C18—H18A	109.5	N21—C28—H28A	109.5
N11—C18—H18B	109.5	N21—C28—H28B	109.5
H18A—C18—H18B	109.5	H28A—C28—H28B	109.5
N11—C18—H18C	109.5	N21—C28—H28C	109.5
H18A—C18—H18C	109.5	H28A—C28—H28C	109.5
H18B—C18—H18C	109.5	H28B—C28—H28C	109.5
N11—C19—H19A	109.5	N21—C29—H29A	109.5
N11—C19—H19B	109.5	N21—C29—H29B	109.5
H19A—C19—H19B	109.5	H29A—C29—H29B	109.5
N11—C19—H19C	109.5	N21—C29—H29C	109.5
H19A—C19—H19C	109.5	H29A—C29—H29C	109.5
H19B—C19—H19C	109.5	H29B—C29—H29C	109.5
N11—C110—H11A	109.5	N21—C210—H21A	109.5
N11—C110—H11B	109.5	N21—C210—H21B	109.5
H11A—C110—H11B	109.5	H21A—C210—H21B	109.5
N11—C110—H11C	109.5	N21—C210—H21C	109.5
H11A—C110—H11C	109.5	H21A—C210—H21C	109.5
H11B—C110—H11C	109.5	H21B—C210—H21C	109.5
C18—N11—C110	109.4 (7)	C21—N21—C210	107.8 (5)
C18—N11—C19	109.2 (6)	C21—N21—C28	111.5 (5)
C110—N11—C19	108.8 (6)	C210—N21—C28	108.0 (6)
C18—N11—C11	110.4 (5)	C21—N21—C29	112.2 (6)
C110—N11—C11	108.0 (6)	C210—N21—C29	107.8 (6)
C19—N11—C11	111.0 (6)	C28—N21—C29	109.4 (6)
N11—C11—C12	114.8 (5)	N21—C21—C22	114.3 (5)
N11—C11—H11D	108.6	N21—C21—H21D	108.7
C12—C11—H11D	108.6	C22—C21—H21D	108.7
N11—C11—H11E	108.6	N21—C21—H21E	108.7
C12—C11—H11E	108.6	C22—C21—H21E	108.7
H11D—C11—H11E	107.5	H21D—C21—H21E	107.6
C11—C12—C13	108.9 (5)	C23—C22—C21	108.9 (5)
C11—C12—H12A	109.9	C23—C22—H22A	109.9
C13—C12—H12A	109.9	C21—C22—H22A	109.9
C11—C12—H12B	109.9	C23—C22—H22B	109.9
C13—C12—H12B	109.9	C21—C22—H22B	109.9
H12A—C12—H12B	108.3	H22A—C22—H22B	108.3
N12—C13—C12	112.2 (5)	N22—C23—C22	111.3 (5)
N12—C13—H13A	109.2	N22—C23—H23A	109.4
C12—C13—H13A	109.2	C22—C23—H23A	109.4
N12—C13—H13B	109.2	N22—C23—H23B	109.4
C12—C13—H13B	109.2	C22—C23—H23B	109.4
H13A—C13—H13B	107.9	H23A—C23—H23B	108.0
C14—N12—C13	121.5 (5)	C24—N22—C23	122.7 (5)
C14—N12—H12N	127 (5)	C24—N22—H22N	118 (4)
C13—N12—H12N	111 (5)	C23—N22—H22N	119 (4)
O11—C14—N12	122.4 (6)	O21—C24—N22	121.9 (5)
O11—C14—C15	121.4 (5)	O21—C24—C25	121.6 (5)
N12—C14—C15	116.2 (5)	N22—C24—C25	116.5 (5)
C16—C15—C17	122.4 (6)	C26—C25—C27	122.2 (6)
C16—C15—C14	117.4 (6)	C26—C25—C24	117.8 (5)
C17—C15—C14	120.2 (5)	C27—C25—C24	120.1 (5)
C15—C16—H16A	120.0	C25—C26—H26A	120.0
C15—C16—H16B	120.0	C25—C26—H26B	120.0
H16A—C16—H16B	120.0	H26A—C26—H26B	120.0
C15—C17—H17A	109.5	C25—C27—H27A	109.5
C15—C17—H17B	109.5	C25—C27—H27B	109.5
H17A—C17—H17B	109.5	H27A—C27—H27B	109.5
C15—C17—H17C	109.5	C25—C27—H27C	109.5
H17A—C17—H17C	109.5	H27A—C27—H27C	109.5
H17B—C17—H17C	109.5	H27B—C27—H27C	109.5
O12—S1—O14	111.7 (4)	O24—S2—O22	111.7 (4)
O12—S1—O13	111.6 (4)	O24—S2—O23	114.4 (5)
O14—S1—O13	113.4 (5)	O22—S2—O23	111.5 (4)
O12—S1—C111	107.7 (3)	O24—S2—C211	107.8 (4)
O14—S1—C111	108.1 (4)	O22—S2—C211	107.2 (3)
O13—S1—C111	103.8 (3)	O23—S2—C211	103.6 (3)
C112—C111—S1	119.0 (5)	C212—C211—S2	119.0 (5)
C112—C111—H11F	107.6	C212—C211—H21F	107.6
S1—C111—H11F	107.6	S2—C211—H21F	107.6
C112—C111—H11G	107.6	C212—C211—H21G	107.6
S1—C111—H11G	107.6	S2—C211—H21G	107.6
H11F—C111—H11G	107.0	H21F—C211—H21G	107.0
N13—C112—C113	106.7 (5)	N23—C212—C214	110.1 (5)
N13—C112—C114	109.7 (4)	N23—C212—C213	106.0 (5)
C113—C112—C114	108.7 (5)	C214—C212—C213	109.0 (5)
N13—C112—C111	111.7 (5)	N23—C212—C211	112.3 (5)
C113—C112—C111	112.5 (5)	C214—C212—C211	107.3 (5)
C114—C112—C111	107.5 (5)	C213—C212—C211	112.3 (5)
C112—C113—H11H	109.5	C212—C213—H21H	109.5
C112—C113—H11I	109.5	C212—C213—H21I	109.5
H11H—C113—H11I	109.5	H21H—C213—H21I	109.5
C112—C113—H11J	109.5	C212—C213—H21J	109.5
H11H—C113—H11J	109.5	H21H—C213—H21J	109.5
H11I—C113—H11J	109.5	H21I—C213—H21J	109.5
C112—C114—H11K	109.5	C212—C214—H21K	109.5
C112—C114—H11L	109.5	C212—C214—H21L	109.5
H11K—C114—H11L	109.5	H21K—C214—H21L	109.5
C112—C114—H11M	109.5	C212—C214—H21M	109.5
H11K—C114—H11M	109.5	H21K—C214—H21M	109.5
H11L—C114—H11M	109.5	H21L—C214—H21M	109.5
C115—N13—C112	126.5 (5)	C215—N23—C212	125.8 (5)
C115—N13—H13N	123 (4)	C215—N23—H23N	112 (5)
C112—N13—H13N	110 (4)	C212—N23—H23N	122 (5)
O15—C115—N13	124.2 (6)	O25—C215—N23	124.2 (6)
O15—C115—C116	121.7 (5)	O25—C215—C216	122.6 (5)
N13—C115—C116	114.1 (5)	N23—C215—C216	113.2 (5)
C117—C116—C115	123.5 (6)	C217—C216—C215	123.2 (6)
C117—C116—H116	118.3	C217—C216—H216	118.4
C115—C116—H116	118.3	C215—C216—H216	118.4
C116—C117—H11N	120.0	C216—C217—H21N	120.0
C116—C117—H11O	120.0	C216—C217—H21O	120.0
H11N—C117—H11O	120.0	H21N—C217—H21O	120.0
			
C18—N11—C11—C12	−74.4 (7)	C210—N21—C21—C22	164.9 (6)
C110—N11—C11—C12	165.9 (6)	C28—N21—C21—C22	−76.7 (7)
C19—N11—C11—C12	46.8 (9)	C29—N21—C21—C22	46.4 (8)
N11—C11—C12—C13	175.5 (5)	N21—C21—C22—C23	176.3 (5)
C11—C12—C13—N12	164.4 (5)	C21—C22—C23—N22	163.8 (5)
C12—C13—N12—C14	85.9 (6)	C22—C23—N22—C24	90.1 (7)
C13—N12—C14—O11	−0.2 (8)	C23—N22—C24—O21	0.5 (9)
C13—N12—C14—C15	179.8 (5)	C23—N22—C24—C25	179.7 (5)
O11—C14—C15—C16	3.7 (9)	O21—C24—C25—C26	0.8 (9)
N12—C14—C15—C16	−176.4 (6)	N22—C24—C25—C26	−178.5 (6)
O11—C14—C15—C17	−175.4 (7)	O21—C24—C25—C27	−179.3 (6)
N12—C14—C15—C17	4.5 (9)	N22—C24—C25—C27	1.4 (9)
O12—S1—C111—C112	65.6 (6)	O24—S2—C211—C212	−56.9 (7)
O14—S1—C111—C112	−55.3 (7)	O22—S2—C211—C212	63.5 (6)
O13—S1—C111—C112	−176.0 (6)	O23—S2—C211—C212	−178.5 (6)
S1—C111—C112—N13	−52.7 (7)	S2—C211—C212—N23	−52.1 (7)
S1—C111—C112—C113	67.2 (7)	S2—C211—C212—C214	−173.1 (5)
S1—C111—C112—C114	−173.1 (5)	S2—C211—C212—C213	67.2 (7)
C113—C112—N13—C115	−177.8 (5)	C214—C212—N23—C215	66.0 (7)
C114—C112—N13—C115	64.6 (7)	C213—C212—N23—C215	−176.3 (5)
C111—C112—N13—C115	−54.5 (7)	C211—C212—N23—C215	−53.4 (8)
C112—N13—C115—O15	−2.6 (9)	C212—N23—C215—O25	−4.1 (9)
C112—N13—C115—C116	177.6 (5)	C212—N23—C215—C216	177.1 (5)
O15—C115—C116—C117	−12.6 (10)	O25—C215—C216—C217	−11.3 (10)
N13—C115—C116—C117	167.2 (6)	N23—C215—C216—C217	167.5 (6)

### Hydrogen-bond geometry (Å, º)

**Table:**

*D*—H···*A*	*D*—H	H···*A*	*D*···*A*	*D*—H···*A*
N12—H12*N*···O15	0.84 (7)	2.02 (7)	2.841 (6)	167 (7)
N13—H13*N*···O11^i^	0.88 (7)	2.10 (7)	2.943 (6)	162 (6)
N22—H22*N*···O25	0.93 (7)	2.00 (7)	2.865 (6)	154 (6)
N23—H23*N*···O21^ii^	0.82 (7)	2.15 (7)	2.961 (6)	174 (7)
C11—H11*D*···O12	0.99	2.31	3.216 (8)	151
C12—H12*A*···O14^iii^	0.99	2.68	3.583 (8)	151
C13—H13*B*···O12	0.99	2.69	3.463 (8)	135
C18—H18*C*···O14^iii^	0.98	2.23	3.182 (10)	164
C18—H18*B*···O22^iii^	0.98	2.25	3.192 (8)	160
C18—H18*A*···O23^iv^	0.98	2.28	3.226 (9)	162
C19—H19*A*···O24^iii^	0.98	2.63	3.555 (10)	157
C110—H11*B*···O21	0.98	2.65	3.182 (11)	114
C116—H116···O11^i^	0.95	2.68	3.375 (7)	131
C117—H11*N*···N12	0.95	2.73	3.338 (8)	123
C21—H21*D*···O22	0.99	2.34	3.236 (8)	151
C22—H22*B*···O24^iv^	0.99	2.53	3.463 (8)	157
C23—H23*B*···O22	0.99	2.65	3.442 (7)	137
C28—H28*A*···O12	0.98	2.20	3.162 (9)	166
C28—H28*B*···O13^v^	0.98	2.27	3.195 (9)	158
C29—H29*C*···O23	0.98	2.41	3.377 (10)	169
C211—H21*F*···O21^i^	0.99	2.71	3.674 (8)	164
C216—H216···O21^ii^	0.95	2.69	3.405 (7)	132

Symmetry codes: (i) *x*−1/2, −*y*+1, *z*; (ii) *x*−1/2, −*y*+2, *z*; (iii) *x*+1/2, −*y*+2, *z*; (iv) *x*+1/2, −*y*+1, *z*; (v) *x*, *y*−1, *z*.
